# Investigating the Atherogenic Risk of Lipoprotein(a) in Type 2 Diabetic Patients

**DOI:** 10.7759/cureus.3030

**Published:** 2018-07-23

**Authors:** Jagannadha R Peela, Omar B Latiwesh, Farag Elshaari, Azhar Hussain, Elsa Tabrez, Emily Viglianco, Ajené Edwards, Farwa Ali, Avinash K Rawal

**Affiliations:** 1 Genetics, St Matthew's University School of Medicine, Grand Cayman, CYM; 2 Medical Laboratory, Higher Institute of Medical Professions, Benghazi, LBY; 3 Biochemistry, Higher Institute of Medical Professions, Benghazi, LBY; 4 Medicine, Xavier University School of Medicine, Oranjestad, ABW; 5 Medicine, St. Matthew’s University School of Medicine, George Town, CYM; 6 Medicine, American University of Antigua College of Medicine, New York, USA; 7 Biochemistry, St. Matthew's University School of Medicine, Grand Cayman, CYM

**Keywords:** lipoprotein(a), type 2 diabetes mellitus, cardiovascular disease, hyperglycemia, diabetic dyslipidemia

## Abstract

Type 2 diabetes mellitus (T2DM) has high morbidity and results in increased risk of mortality mainly due to cardiovascular diseases. Different factors have been found to be responsible for the increased prevalence of coronary artery disease (CAD) in T2DM. One of these factors includes raised serum levels of lipoprotein(a) (Lp(a)). The present study was designed to evaluate the association of Lp(a) levels with T2DM in Libyan patients and find the degree of association between Lp(a), glycemic control, insulin, and lipid profile. The study included 100 T2DM patients, recruited from the Benghazi Center for Diagnosis and Treatment of Diabetes, and 30 apparently healthy age and sex-matched individuals, to serve as controls. All participants completed a questionnaire to obtain clinical information and medical history. Blood samples were collected and analyzed for Lp(a), fasting blood glucose (FBS), HbA1c, insulin, total cholesterol (TC), triglycerides (TAG), low-density lipoprotein c (LDL-c), and high-density lipoprotein c (HDL-c). The results from the comparison between the control and experimental groups showed that Lp(a) was significantly higher in diabetic patients. It showed the positive correlation with TC and LDL-c. On the contrary, it showed no significant correlations with glycemic control parameters nor insulin, TAG, HDL-c, body mass index (BMI), and blood pressor (BP). Cardiovascular disease (CVD) risk in type 2 diabetic patients could be dependent on risk factors other than LDL-c, which may not be an independent risk factor for the development and progression of atherogenesis in T2DM. Lp(a) may be a new metabolic syndrome risk factor, and it may be useful as a cardiovascular risk biomarker in future clinical practice.

## Introduction

Type 2 diabetes mellitus (T2DM) is a well-recognized risk factor for cardiovascular disease (CVD), cerebrovascular disease, and peripheral vascular disease, aggravating the effects of other common risk factors, such as smoking, hypertension, and hypercholesterolemia [1,2]. The mortality associated with cardiovascular diseases in people with diabetes mellitus is significantly higher than the mortality in nondiabetic individuals [3]. The increased risk of atherosclerosis in diabetics consists of multiple factors. Diabetic dyslipidemia, which is characterized by high plasma triacylglycerol concentration, low high-density lipoprotein (HDL) cholesterol concentration, and increased concentration of small, dense low-density lipoprotein (LDL) particles, are among the key factors that increase the risk of CVD in diabetics [4].

Lipoprotein(a) (Lp(a)) was described nearly 50 years ago by Kare Berg [5] and has been considered to be a genetic variant of low-density lipoprotein. Later, it was recognized as a distinct class of lipoproteins [6]. In many studies elevated plasma Lp(a) levels have been shown to contribute significantly to the incidence of CVD [7].

Lp(a) consists of an LDL-like core lipoprotein covalently linked to glycoprotein apolipoprotein(a) (Apo(a)) [8]. The protease domain of Apo(a) contains 88% amino acid identity to the protease domain of plasminogen but is not an active protease able to degrade fibrin [9]. This homology prevents the activation of plasminogen by tissue plasminogen activator (t-PA) [10], partly by competing with plasminogen for binding to the fibrin or endothelial cell surface [11], and also by stimulating endothelial cell synthesis of plasminogen activator inhibitor (PAI-1) possibly hindering fibrinolysis [12]. Moreover, lipoprotein(a) has been demonstrated to bind and inhibit tissue factor pathway inhibitor (TFPI), a potent inhibitor of the tissue factor-mediated coagulation cascade, thus perhaps directly promoting thrombosis [13].

The association of lipoprotein(a) with T2DM is controversial. A study conducted by Ramirez et al. [14], found a raised mean Lp(a) level in type 2 diabetic patients but found no association of glycosylated hemoglobin with Lp(a) in diabetics. On the contrary, a cross-sectional analysis of plasma Lp(a) concentrations from 36 studies reported that Lp(a) levels were 11% lower in diabetic patients than in normal subjects [15]. Furthermore, another study showed no differences in serum Lp(a) levels between diabetic patients and controls [16]. Our present study was undertaken to estimate serum Lp(a) levels in Libyan patients with type 2 diabetes, and find out its association with glycemic control, lipid profile, and serum insulin.

## Materials and methods

The present case-control study was conducted during the period of September 2013 to March 2014 and included 100 type 2 diabetic patients recruited from the Benghazi Center for Diagnosis and Treatment of Diabetes and 30 apparently healthy, age, and sex-matched individuals selected from the Higher Institute of Medical Professions-Benghazi to serve as controls. Before the study, informed consent was obtained from all participants, and the study design was approved by the Ethics Review Board of the University of Benghazi. The patient’s diagnoses were based on the American Diabetes Association criteria 2006 (i.e., A1c ≥ 6.5%, or fasting blood glucose (FBS) level ≥ 126 mg/dL or 2-h plasma glucose ≥ 200 mg/dl during an oral glucose tolerance test). All participants completed a survey to obtain relevant information such as age, sex, diet, activity level, history of smoking, chronic medical conditions, and medications. Each participant’s height and weight were measured, and body mass index (BMI) was calculated by dividing the weight in kilograms by height in meters squared. Blood pressure (BP) was measured using mercury sphygmomanometer.

At the onset of the study, all patients presented with stable metabolic conditions. Patients excluded from the study were those suffering from any disease that could affect their metabolic status and the parameters studied, including nephrotic syndrome, acute or chronic renal failure, liver disease, thyroid disorders, acute infections, stroke, gout, diabetic ketoacidosis, and non-ketotic hyperosmolar syndrome, as well as those with a history of familial hypercholesterolemia or acute myocardial infarction. Patients who recorded taking insulin, lipid-lowering agents, oral contraceptives, calcium antagonist, beta blockers, and steroids were also excluded from the study. The control group consisted of healthy subjects who were not suffering from an acute infection or metabolic or psychological disorder. They were non-smoker, non-overweight, and had no history of familial hypercholesterolemia or diabetes mellitus (DM).

Venous blood samples were drawn from all the participants after at least 10 hours of fasting. Blood was collected in ethylenediaminetetraacetic acid (EDTA) and plain tubes, and serum was separated from plain tubes and stored at −70°C until the assays were performed. The whole blood was stored at 4–8°C and analyzed for hemoglobin A1c (HbA1c) within a week. The measurements of glucose, HbA1c, total cholesterol (TC), triglycerides (TAG), HDL, and LDL were conducted using the standard procedures and available commercial kits in a fully automated system COBAS INTEGRA 400 plus (ROCH, Germany). Serum insulin was measured by electrochemiluminescence using a fully automated COBAS e 411 (ROCH, Germany). Serum Lp(a) was estimated by Sandwich ELISA using kits supplied by Abcam, USA. The diabetic patients were divided into good and poor glycemic control groups based on a cut-off HbA1c value of 7.5%.

The data were analyzed using SPSS version 17 (SPSS IBM, Inc., Chicago, IL). Descriptive characteristics of the study participants were calculated as the mean ± standard deviation (SD). Differences in various parameters between subject groups and degree of association between Lp(a) and some clinical and biochemical parameters were found using Analysis of Variance (ANOVA) and Pearson’s correlation with the p-value less than 0.05 considered as statistically significant.

## Results

Clinical characteristics and glycemic status of diabetic patients and healthy control subjects are shown in Table 1. BMI was significantly higher in both groups of diabetics compared to the normal control group (p < 0.05). There was a significant difference in BMI between diabetics with good glycemic control and those with poor glycemic control (p = 0.009). Additionally, systolic and diastolic blood pressure measurements showed significant differences between diabetic patients and the control group (p < 0.05). Non-significant differences in systolic and diastolic blood pressure measurements were found when diabetics with good glycemic control compared to diabetics with poor glycemic control (p = 0.87 and p = 0.88, respectively). Diabetic patients had significantly higher blood glucose, HbA1c, and insulin than non-diabetic control subjects (p < 0.05). Likewise, diabetics with poor glycemic control had significantly higher levels of blood glucose when compared to diabetics with good glycemic control (p = 0.00). On the other hand, the difference in serum insulin between diabetics with good and poor glycemic control was statistically insignificant (p = 0.19).

**Table 1:** Clinical characteristics and glycemic status of type 2 diabetes mellitus (T2DM) patients with poor glycemic control (HbA1c > 7.5%), and good glycemic control (HbA1c ≤ 7.5%), and control subjects. N: Number of subjects *: Data are expressed as mean ± Standard Deviation (SD)

Groups	Diabetes Mellitus patients with HbA1c > 7.5%; (N = 54)	Diabetes Mellitus patients with HbA1c ≤ 7.5%; (N = 46)	Control (N = 30)	p-Value
Age (years)	49.39 ± 4.81*	51.21 ± 5.48	47.33 ± 6.18	-
Gender M/F	26/31	19/24	14/16	-
Body Mass Index (BMI) (Kg/m^2^)	26.8 ± 0.88	26.34 ± 1.03	24.48 ± 0.32	0.00
Diastolic Blood Pressure (BP) (mm Hg)	84.65 ± 4.75	84.78 ± 4.48	76.09 ± 3.7	0.00
Systolic Blood Pressure (BP) (mm Hg)	132.72 ± 8.36	132.98 ± 8.1	114.1 ± 6.83	0.00
Fasting Blood Glucose (FBS) (mg/dl)	185.1 ± 62.7	129.93 ± 38	89.4 ± 5.56	0.00
Hemoglobin A1c (HbA1c) (%)	9.48 ± 1.3	6.41 ± 0.73	4.88 ± 0.27	0.00
Insulin (µIU/ml)	16.11 ± 11	13.54 ± 10.3	8.32 ± 4.2	0.003

Lipid profile and Lp(a) levels of diabetic and control subjects are shown in Table 2. The mean levels of serum TC, TAG, low-density lipoprotein-c (LDL-c), and LDL-c/high-density lipoprotein-c (HDL-c) ratio were significantly higher in both groups of diabetic patients compared to control subjects (p < 0.05). Dissimilarly, comparing serum TC, LDL-c, and LDL-c/HDL-c ratio of diabetics with good glycemic control to those of diabetics with poor glycemic control showed no significant differences (p = 0.64, p = 0.42, and p = 0.56, respectively). There was a significant difference in TAGs when diabetics with good glycemic control and poor glycemic control were compared (p = 0.003). On the contrary, the HDL-c concentration was significantly lower in diabetics with good and poor glycemic control than the concentrations of control subjects (p < 0.05). The comparison showed a non-significant difference in HDL-c between diabetic groups with poor glycemic control and good glycemic control (p = 0.28).

As shown in Table 2, the mean concentration of Lp(a) in both groups of type 2 diabetic patients is significantly higher than that of the normal control group (p < 0.05). Lp(a) levels are insignificantly lower in diabetic patients with poor glycemic control when compared to the patients with good glycemic control (p = 0.30). The mean Lp(a) levels in both male and female diabetics were comparable (males vs females- 11.3 mg/dl vs 12.2 mg/dl, p = 0.29). In addition, no significant differences were observed in Lp(a) levels in relation to age or duration of disease.

**Table 2:** Lipid profile and lipoprotein(a) levels in diabetic and control subjects. N: Number of subjects *: Data are expressed as mean ± Standard Deviation (SD)

Groups	Diabetes Mellitus patients with HbA1c > 7.5%; (N = 54)	Diabetes Mellitus patients with HbA1c ≤ 7.5%; (N = 46)	Control (N = 30)	p-Value
Total Cholesterol (TC) (mg/dl)	225.61 ± 44*	222.39 ± 31.34	163 ± 17.56	0.00
Triglycerides (TAG) (mg/dl)	205.62 ± 83.53	159.21 ± 78.24	109.5 ± 31.4	0.00
Low-density lipoprotein-c (LDL-c) (mg/dl)	139.53 ± 43.67	142.97 ± 33.88	91.51 ± 14.25	0.00
High-density lipoprotein-c (HDL-c) (mg/dl)	44.69 ± 11.37	46.83 ± 9.86	70.18 ± 6.77	0.00
Low-density lipoprotein (LDL)/High-density lipoprotein (HDL) Ratio	3.29 ± 1.27	3.17 ± 0.98	1.31 ± 0.26	0.00
Lipoprotein(a) (Lp(a)) (mg/dl)	11.4 ± 4.2	12.25 ± 4.9	8.13 ± 2	0.00

**Figure 1:**
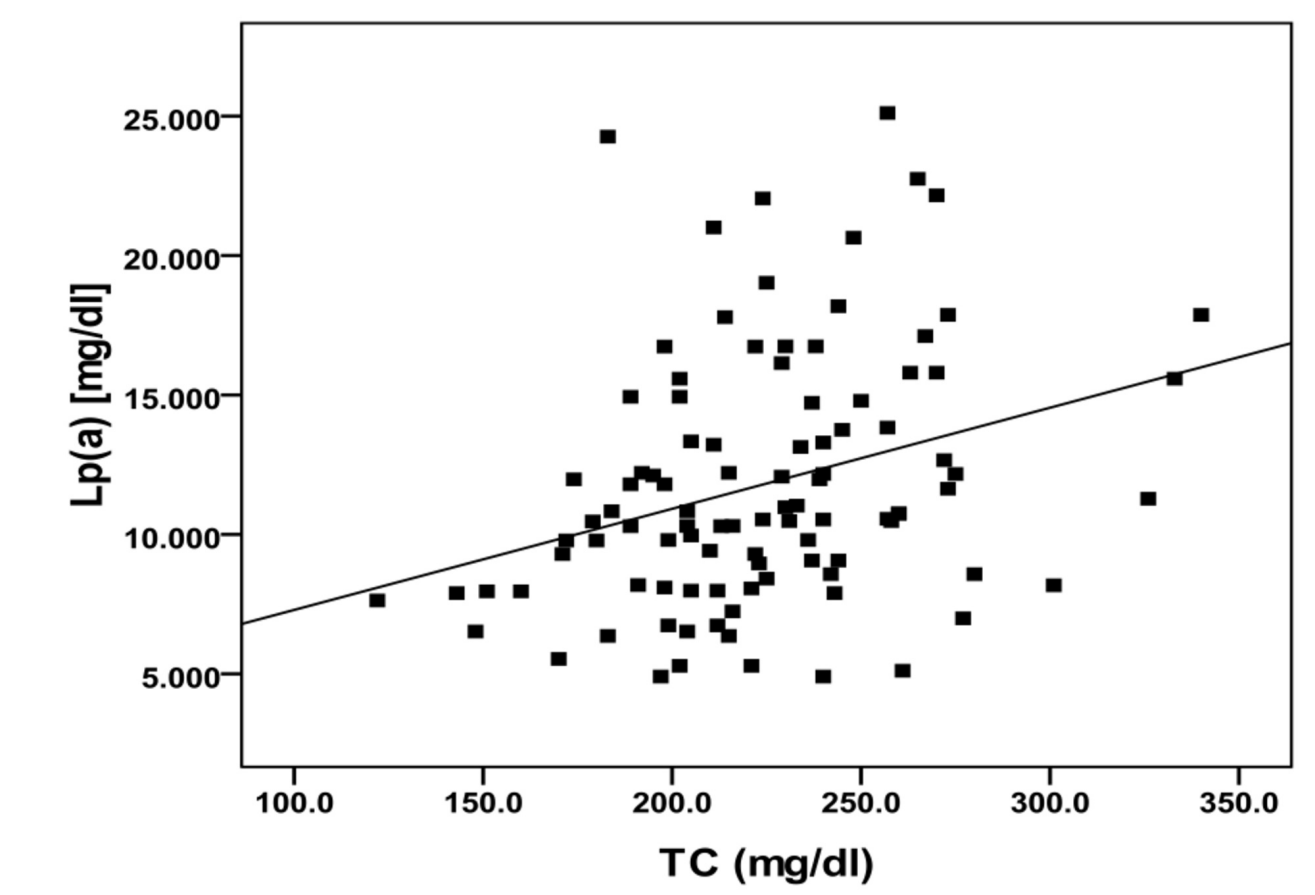
Correlation between lipoprotein(a) and total cholesterol in diabetic group. TC: Total cholesterol; Lp(a): Lipoprotein(a).

**Figure 2:**
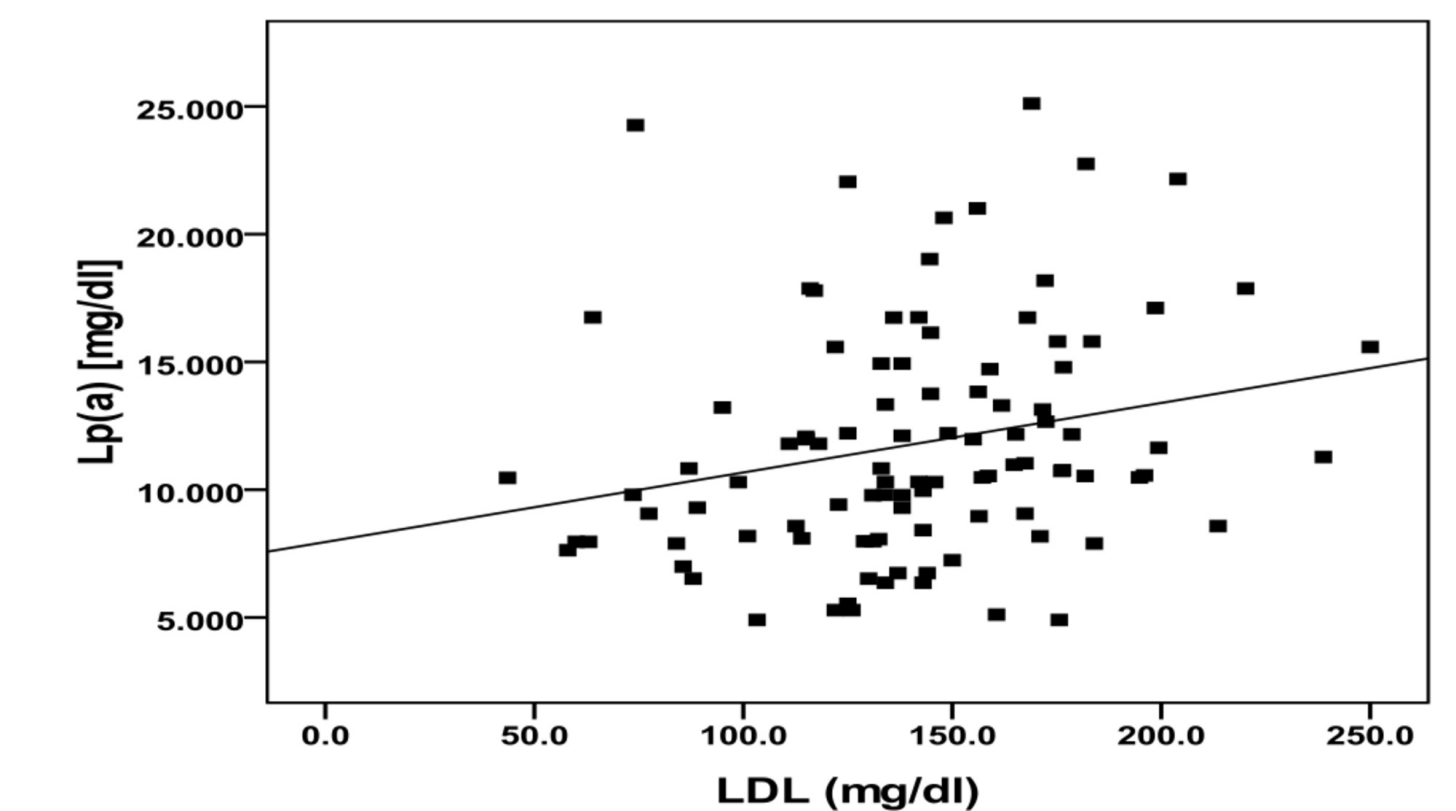
Correlation between lipoprotein(a) and low-density lipoprotein in diabetic group. Lp(a): Lipoprotein(a); LDL: Low-density lipoprotein.

A summary of the correlations among LP(a) and some clinical and biochemical parameters in diabetic patients is demonstrated in Table 3. In diabetic patients, Lp(a) was positively correlated with TC and LDL-c, and the correlation was statistically significant (Figures 1, 2). However, no significant correlations were seen between Lp(a) and, age, BMI, BP, TAG, HDL-c, LDL-c/HDL-c ratio, insulin and glycemic control parameters (FBS and HbA1c).

**Table 3:** Pearson correlation (r) between lipoprotein(a) and some clinical and biochemical parameters in type 2 diabetic patients. **: Correlation is significant at the 0.01 level. *: Correlation is significant at the 0.05 level.

Variable	Pearson correlation (r)	p-Value
Age	0.154	0.127
Body Mass Index (BMI)	-0.044	0.663
Systolic Blood Pressure (BP)	0.051	0.613
Diastolic Blood Pressure (BP)	0.103	0.306
Fasting Blood Glucose (FBS)	0.025	0.802
Hemoglobin A1c (HbA1c)	-0.001	0.990
Total Cholesterol (TC)	0.309**	0.002
High-density lipoprotein-c (HDL-c)	0.042	0.680
Low-density lipoprotein-c (LDL-c)	0.236*	0.018
Triglycerides (TAG)	-0.013	0.900
Insulin	0.040	0.694
Low-density lipoprotein-c (LDL-c)/High-density lipoprotein-c (HDL-c)	0.163	0.105

## Discussion

The major finding of the present study was a significantly higher level of Lp(a) in diabetic patients when compared with control subjects. This significant finding is consistent with results reported by several studies [17-20]. The effect of hyperglycemia on the rate of Apo(a) synthesis is still not exactly known. It is evident from many studies that glycosylation prolongs the half-life of lipoproteins. This may be applicable for Lp(a) where many studies have shown diabetic patients have an increased concentration of glycosylated hemoglobin, which may contribute to their higher level of Lp(a) [21-22].

Plasma Lp(a) concentrations are primarily regulated at the level of the Apo(a) gene, and an inverse relationship was found between plasma Lp(a) concentration and Apo(a) size which may result from an inefficient secretion of larger Apo(a) isoforms from the hepatocytes [23]. A study of Ribault et al. [24], found that type 2 diabetic patients have a higher expression of low molecular weight isoforms of Apo(a), raising the possibility of which diabetic patients in the present study may have small size isoforms of Apo(a), resulting in higher levels of Lp(a).

Studies of Chang et al. and Chico et al. reported no differences in serum Lp(a) levels between diabetic patients and controls [16,25]. Rainwater et al. and Hernandez et al. demonstrated lower serum Lp(a) levels in diabetic patients when compared to control subjects. Rainwater et al. attributed their findings to the presumption, nonenzymatic glycosylation of Apo(a) in type 2 diabetic patients increases the size of the molecule, which may be responsible for lower plasma Lp(a) levels in type 2 diabetics as there is inverse relationship between Apo(a) size and plasma Lp(a) [26,27].

No significant correlation has been shown between either fasting glucose or HbA1c and plasma Lp(a). Changes in Lp(a) concentrations relative to glycemic control remain controversial; however, most studies have failed to show any correlation between Lp(a) levels and glycemic control in patients with T2DM [14,28].

In observations, Lp(a) was positively correlated with LDL-c and total cholesterol but insignificantly correlated to TAG, HDL-c, BMI, or BP. These findings are in agreement with the results of many studies [18,29]. Apo(a) is predominantly synthesized in the liver and enters the circulation, where the LDL moiety is attached [30]. Thus, in diabetic dyslipidemia, there is a higher number of LDL particles present in the circulation, and there would be more union between LDL and Apo(a) and therefore, higher concentrations of Lp(a). Reasons for exclusion were myocardial infarction in the previous year, current angina or heart failure, accelerated hypertension, proliferative or pre-proliferative retinopathy, renal failure with a plasma creatinine concentration >175 μmol/l, other life-threatening disease such as cancer, an illness requiring systemic steroids, an occupation which precluded insulin treatment, language difficulties, or ketonuria >3 mmol/l suggestive of insulin dependent diabetes mellitus [18,28].

A study of Ogbera and Azenabor observed a negative correlation between Lp(a) and TAGs [28], explaining this relation by stating that Apo(a) is present in triacylglycerol-rich particles (TRPs). These Apo(a)-containing TRPs, in parallel with chylomicron remnants, would be rapidly endocytosed by the liver through the remnant-receptor pathway. Thus, the lower levels of Lp(a) in patients with hypertriacylglycerolemia could be the result of the rapid catabolism of TRP- Apo(a) [27]. Supportive evidence in favor of this finding was reported by which improving insulin sensitivity in T2DM subjects by rosiglitazone (insulin-sensitizing agent) was associated with decreasing of triacylglycerols and increasing of Lp(a) concentration [29,30].

Some studies revealed a negative relationship between Lp(a) and serum insulin; studies speculated that higher Lp(a) levels among patients with a longer duration of type 2 diabe­tes may be attributed to lower plasma insulin levels in individuals. An in vitro study showed that insulin suppresses Apo(a) production in primary cynomolgus monkey hepatocytes [19]. This finding is inconsistent with our study, which showed a non-significant association between Lp(a) and serum insulin.

The association of Lp(a) levels in DM has been a matter of some controversies. The major reasons for the inconsistent results of the prospective studies have been attributed to the variations in study design. Collection and storage of samples, analytical techniques used, and methods used for statistical analysis and population differences reflect the known ethnic variability in the Apo(a) size isoforms and distribution of plasma Lp(a) levels.

## Conclusions

CVD risk in type 2 diabetic patients could be dependent on additional lipid risk factors and LDL-c. This may not be an independent risk factor for the development and progression of atherogenesis in type 2 diabetes mellitus. This study concluded that Lp(a) values may be an independent predictive risk factor in type 2 diabetes mellitus patients for the development of cardiovascular disease. Using Lp(a) levels as a screening tool could prove to be a useful biomarker for detecting those patients who are at high risk of CVD. Lp(a) may be a new member of metabolic syndrome and it may be useful in routine clinical practice in the future as a biomarker for determining cardiovascular risk.

## References

[1] Stamler J, Vaccaro O, Neaton JD, Wentworth D (1993). Diabetes, other risk factors, and 12-yr cardiovascular mortality for men screened in the Multiple Risk Factor Intervention Trial. Diabetes Care Feb.

[2] Almdal T, Scharling H, Jensen JS, Vestergaard H (2004). The independent effect of type 2 diabetes mellitus on ischemic heart disease, stroke, and death: a population-based study of 13 000 men and women with 20 years of follow-up. JAMA Intern Med.

[3] Sprafka JM, Burke GL, Folsom AR, McGovern PG, Hahn HP (1991). Trends in prevalence of diabetes mellitus in patients with myocardial infarction and effect of diabetes on survival: the Minnesota Heart Survey. Diabetes Care.

[4] Hachem SB, Mooradian AD (2006). Familial dyslipidaemias: an overview of genetics, pathophysiology and management. Drugs.

[5] Berg K (1963). A new serum type system in man--the LP system. Acta Pathol Microbiol Scand.

[6] Dubé JB, Boffa MB, Hegele RA, Koschinsky ML (2012). Lipoprotein(a): more interesting than ever after 50 years. Curr Opin Lipidol.

[7] Marcovina SM, Koschinsky ML, Albers JJ, Skarlatos S (2003). Report of the National Heart Lung, and Blood Institute workshop on lipoprotein(a) and cardiovascular disease: recent advances and future directions. Clin Chem.

[8] Gries A, Nimpf J, Nimpf M, Wurm H, Kostner GM (1987). Free and Apo B-associated Lpa-specific protein in human serum. Clinica Chimica Acta.

[9] McLean JW, Tomlinson JE, Kuang WJ (1987). cDNA sequence of human apolipoprotein(a) is homologous to plasminogen. Nature.

[10] Boffa MB, Koschinsky ML (2016). Lipoprotein (a): truly a direct prothrombotic factor in cardiovascular disease?. J Lipid Res.

[11] Loscalzo J, Weinfeld M, Fless GM, Scanu AM (1990). Lipoprotein(a), fibrin binding, and plasminogen activation. Arterioscler Thromb Vasc Biol.

[12] Etingin OR, Hajjar DP, Hajjar KA, Harpel PC, Nachman RL (1991). Lipoprotein (a) regulates plasminogen activator inhibitor-1 expression in endothelial cells. A potential mechanism in thrombogenesis. J Biol Chem.

[13] Caplice NM, Panetta C, Peterson TE (2001). Lipoprotein (a) binds and inactivates tissue factor pathway inhibitor: a novel link between lipoproteins and thrombosis. Blood.

[14] Ramirez LC, Arauz-Pacheco C, Lackner C, Albright G, Adams BV, Raskin P (1992). Lipoprotein (a) levels in diabetes mellitus: relationship to metabolic control. Ann Intern Med.

[15] The Emerging Risk Factors Collaboration (2009). Lipoprotein (a) concentration and the risk of coronary heart disease, stroke, and nonvascular mortality. JAMA.

[16] Chico A, Pérez A, Caixàs A, Ordoñez J, Pou JM, de Leiva A (1996). Lipoprotein(a) concentrations and non-insulin-dependent diabetes mellitus: relationship to glycaemic control and diabetic complications. Diabetes Res Clin Pract.

[17] Habib SS (2013). Serum lipoprotein (a) and high sensitivity C reactive protein levels in Saudi patients with type 2 diabetes mellitus and their relationship with glycemic control. Turk J Med Sci.

[18] Smaoui M, Hammami S, Chaaba R (2004). Lipids and lipoprotein (a) concentrations in Tunisian type 2 diabetic patients: relationship to glycemic control and coronary heart disease. J Diabetes Complications.

[19] Habib SS, Aslam M, Shah SF, Naveed AK (2009). Lipoprotein (a) is associated with basal insulin levels in patients with type 2 Diabetes Mellitus (Article in English, Portuguese, Spanish). Arq Bras Cardiol.

[20] Patil M, Kumar N, Nusrath A, Jayaram S, Rajeshwari A (2014). Association of HBA1C with serum lipid profile and lipoprotein (A) in type 2 diabetes mellitus. Int J Curr Res Rev.

[21] Klaya F, Durlach V, Bertin E, Monier F, Monboisse J-C, Gillery P (1997). Evaluation of serum glycated lipoprotein (a) levels in noninsulin-dependent diabetic patients. Clin Biochem.

[22] Maca T, Mlekusch W, Doweik L, Budinsky AC, Bischof M, Minar E, Schillinger M (2007). Influence and interaction of diabetes and lipoprotein (a) serum levels on mortality of patients with peripheral artery disease. Eur J Clin Invest.

[23] Marcovina SM, Koschinsky ML (1999). Lipoprotein(a) concentration and apolipoprotein(a) size: a synergistic role in advanced atherosclerosis?. Circulation.

[24] Ribault A, Durou M, Letellier C, Wojcik F, Poirier JY, Ruelland A (2000). Determination of lipoprotein (a) concentrations and apolipoprotein (a) molecular weights in diabetic patients. Diabetes Metab.

[25] Chang CJ, Kao JT, Wu TJ, Lu FH, Tai TY (1995). Serum lipids and lipoprotein (a) concentrations in Chinese NIDDM patients: relation to metabolic control. Diabetes Care.

[26] Rainwater DL, MacCluer JW, Stern MP, VandeBerg JL, Haffner SM (1994). Effects of NIDDM on lipoprotein(a) concentration and apolipoprotein(a) size. Diabetes.

[27] Hernández C, Chacón P, García-Pascual L, Simó R (2001). Differential influence of LDL cholesterol and triglycerides on lipoprotein (a) concentrations in diabetic patients. Diabetes Care.

[28] Ogbera AO, Azenabor AO (2010). Lipoprotein (a), C-reactive protein and some metabolic cardiovascular risk factors in type 2 DM. Diabetol Metab Syndr.

[29] Habib S, Aslam M (2004). Lipids and lipoprotein (a) concentrations in Pakistani patients with type 2 diabetes mellitus. Diabetes Obes Metab.

[30] Ko SH, Song KH, Ahn YB (2003). The effect of rosiglitazone on serum lipoprotein (a) levels in Korean patients with type 2 diabetes mellitus. Metabolism.

